# Toxicity Evaluation of a Novel Magnetic Resonance Imaging Marker, CoCl2-N-Acetylcysteine, in Rats

**DOI:** 10.1155/2018/9173452

**Published:** 2018-12-02

**Authors:** Li Wang, Mihai Gagea, Karen Martirosyan, Mary Johansen, Timothy Madden, Lisa Norberg, Kirk S. Culotta, Steven J. Frank

**Affiliations:** ^1^Department of Experimental Radiation Oncology, The University of Texas MD Anderson Cancer Center, USA; ^2^Departments of Veterinary Medicine & Surgery, The University of Texas MD Anderson Cancer Center, USA; ^3^Department of Physics, University of Texas Rio Grande Valley, USA; ^4^Strategia Therapeutics, Inc., USA; ^5^InPharma, LLC., USA; ^6^Department of Pathology, The University of Texas MD Anderson Cancer Center, USA; ^7^Department of Experimental Therapeutics, The University of Texas MD Anderson Cancer Center, USA; ^8^Merck & Co., Inc., Kenilworth, NJ, USA; ^9^Department of Radiation Oncology, The University of Texas MD Anderson Cancer Center, USA

## Abstract

C4 (cobalt dichloride-N-acetylcysteine [1% CoCl_2_:2% NAC]) is a novel magnetic resonance imaging contrast marker that facilitates visualization of implanted radioactive seeds in cancer brachytherapy. We evaluated the toxicity of C4. Rats were assigned to control (0% CoCl_2_:NAC), low-dose (0.1% CoCl_2_:2% NAC), reference-dose (C4), and high-dose (10% CoCl_2_:2% NAC) groups. Agent was injected into the left quadriceps femoris muscle of the rats. Endpoints were organ and body weights, hematology, and serum chemistry and histopathologic changes of tissues at 48 hours and 28 and 63 days after dosing. Student's *t* tests were used. No abnormalities in clinical signs, terminal body and organ weights, or hematologic and serum chemistry were noted, and no gross or histopathologic lesions of systemic tissue toxicity were found in any treatment group at any time point studied. At the site of injection, concentration-dependent acute responses were observed in all treatment groups at 48 hours after dosing and were recovered by 28 days. No myofiber degeneration or necrosis was observed at 28 or 63 days in any group. In conclusion, a single intramuscular dose of C4 produced no acute or chronic systemic toxicity or inflammation in rats, suggesting that C4 may be toxicologically safe for clinical use in cancer brachytherapy.

## 1. Introduction

The current standard of care after brachytherapy for localized prostate cancer includes an image-guided quality assurance check of the radioactive seed placement and anticipated dose distribution. Historically the imaging technique used for this purpose is computed tomography (CT). Although CT can effectively visualize the seeds, it is less successful for visualizing soft tissues such as the prostate. Magnetic resonance imaging (MRI), on the other hand, is ideal for visualizing soft tissues [1, 2] but requires a positive-contrast marker to identify the seeds [3]; nevertheless, MRI is increasingly being viewed as an excellent imaging tool for posttreatment quality assurance [4, 5]. One positive-contrast marker under T1-weighted sequence scanning being developed for this purpose is C4, a complex of cobalt dichloride and N-acetylcysteine (CoCl_2_:NAC) that is encapsulated within a permanently implantable device [3]. Previous studies have shown that C4 does not affect anisotropy or volumetric dosimetry when placed adjacent to radioactive seeds, nor does it alter the T1 positive-contrast signal after exposure to high doses of radiation [6]. C4 further has a complexation rate of >90%, is stable in solution, and remains stable upon exposure to human plasma at physiological pH and temperature (Supplemental Tables 1 and 2). A study of the pharmacokinetics, biodistribution, and acute toxicity of C4 demonstrated rapid, dual-route elimination and lack of toxicity after injection of the C4 solution into the prostate and periprostatic tissues [7]. However, some evidence of intraprostatic inflammation was observed on histopathologic evaluation; whether that inflammation was related to the surgical procedure or to C4 was unclear. Further, whether leakage of the C4 solution from the capsules after prostate brachytherapy would potentiate systemic inflammatory effects or produce acute or chronic toxicity is unknown. Hence the purpose of this study was to evaluate the potential inflammatory toxicity of C4 in the target organs and systemically. An experimental model in which C4 is injected into the quadriceps femoris muscle of rats, which would allow evaluation of local tissue tolerance and recovery from of any damage, and accurate assessment of local and systemic inflammation was used.

## 2. Materials and Methods

### 2.1. C4 Solutions

Solutions at each dose level were prepared within 1 week of administration under a standard operating procedure in which CoCl_2_:NAC is prepared from cobalt chloride hexahydrate (CoCl_2_ • 6H_2_O; Sigma), N-acetylcysteine (C_5_H_9_NO_3_S; Sigma), and ultrapure, filtered, double-deionized water (Milli-Q, Millipore). Solutions were stored at 5 ± 3°C and protected from light until administration. At the prescribed injection times, solutions were warmed to room temperature and transferred to the animal facility while still protected from light. Solutions were analyzed for total cobalt content both before and after dosing by inductively coupled plasma mass spectrometry at A&B Labs (Houston, TX).

### 2.2. Animals

Sixty male Sprague-Dawley rats, aged 12-13 weeks and weighing 300–450 g, were supplied by Charles River Laboratories. This species has been accepted to support studies of compounds intended to be used in humans. Procedures for animal housing and care were carried out in accordance with the Animal Welfare Act as amended in the recommendations and guidelines of the Public Health Service, the US Department of Agriculture, the Association for Assessment and Accreditation of Laboratory Animal Care International, and Institutional Animal Care and Use Committee of UT MD Anderson Cancer Center. Environmental conditions were within specified limits in at least 90% of scheduled observations. Rats were housed up to 3 per cage with bedding or cage board (rather than cedar or pine chips), and cages were sanitized regularly. No known contaminants that could affect the results of the study were present in the bedding. The rats were fed a commercial, dry rodent chow ad libitum, and water was also given ad libitum. All rats were quarantined for at least 3 days before dosing, and no prophylactic or therapeutic treatments were given during the quarantine period. Rats were identified individually, by cage, and by treatment group.

### 2.3. Animal Treatments

Rats were assigned to one of four dosage groups (15 rats/dose group): control (0% CoCl_2_:NAC), low dose (0.1% CoCl_2_:2% NAC), mid- or reference dose (1% CoCl_2_:2% NAC; this is the solution used in the MRI-marker medical device), or high dose (10% CoCl_2_:2% NAC). These concentrations were based on the concentrations contained within the reference medical device (1% CoCl_2_ • 6H_2_O), bracketed by one higher level (10% CoCl_2_ • 6H_2_O) and one lower level (0.1% CoCl_2_ • 6H_2_O). All solutions contained 2% NAC. The solutions were given in 9-*μ*L aliquots with a Hamilton 1700 series gas-tight 25 *μ*L syringe with a 27 gauge/0.5 inch needle by intramuscular (i.m.) injection into the middle of the left hind limb quadriceps femoris. Rats were anesthetized and maintained with isoflurane inhalation to prevent trauma to the muscle during the injection. Once the rats were anesthetized and immediately before the injection, hair around the injection site area was clipped and the injection site wiped with 70% alcohol. Unless otherwise noted, the rats were injected in the order listed above (control, low dose, reference dose, and high dose). The injection sites were circled immediately after the injection with a permanent marker.

### 2.4. Clinical Observations, Necropsy, and Tissue Sampling

Rats were observed for clinical signs daily and weighed twice weekly. At 2 days (48 hours), 28 days, or 63 days after the injection, 5 rats from each dose group (3 experimental and 1 control) were euthanized with carbon dioxide, weighed, exsanguinated, and subjected to necropsy. Blood samples were collected immediately after euthanasia via cardiac puncture for hematology and serum chemistry analyses from all 4 groups at each of the 3 time points. All rats were weighed just before terminal blood collection. At necropsy, the liver, kidney, and spleen were weighed, all organs were examined grossly, and tissue samples described in Supplemental Table 3 were collected in 10% neutral buffered formalin for fixation. In addition, the entire quadriceps femoris muscle, the target tissue with the site of injection, from the treated left hind limb (containing the marked area of injection site), and the untreated right hind limb from all animals (control and 3 dose groups at 48 hours, 28 days, and 63 days after treatment) were collected in formalin for fixation, histologic processing, and microscopic examination. All remaining tissues from the 63-day time point dose groups were preserved in 10% neutral buffered formalin and archived. A veterinary pathologist examined all animals at necropsy and recorded the presence of tissue changes.

### 2.5. Blood Sample Processing and Analysis

Blood samples collected in microtubes with serum separator were allowed to clot for 30 minutes after collection and were then centrifuged for 2 minutes at 17,000 rpm for isolating the blood serum that was processed for serum chemistry analysis. For hematologic analyses, blood samples were collected in microtubes containing ethylenediaminetetraacetic acid (EDTA); for the complete blood cell count analysis, blood smears were stained with Diff-Quik. Complete blood cell count analysis was performed with a Cobas Integra 400 Plus instrument (Roche) and serum chemistry measurements with an Advia 120 instrument (Siemens AG).

### 2.6. Tissue Processing for Histologic Analysis

After fixation, all tissues (Tables 1 and 2) from the control and high-dose groups euthanized at 48 hours and 28 days after treatment were processed, embedded in paraffin blocks, and cut into 4-*µ*m thick sections that were mounted on glass slides and stained with hematoxylin-and-eosin (H&E) for microscopic examination. The entire left quadriceps femoris muscle, including the injection site, from all rats was cut in multiple slices at 3-mm intervals for similar histologic preparation. All histologic sections of these organs, including the observed gross lesions, were examined and evaluated microscopically by a veterinary pathologist. All histologic sections of quadriceps femoris muscle from treated left hind limbs were scanned with Aperio, and the extent of lesions at the site of i.m. injection was measured in all sections.

### 2.7. Statistical Analysis

Each experiment was repeated at least three times. Unless otherwise noted, data are presented as means and ranges, and Student's *t* tests (unpaired, unequal variance) were used to compare two groups of independent samples for body weight, absolute and relative organ weights, and blood hematologic or serum chemistry values. A* P* value of <0.05 was considered to indicate statistically significant differences.

## 3. Results

### 3.1. Clinical Observations

All animals survived until their scheduled termination dates. No adverse clinical signs or abnormalities in body weight or in absolute or relative organ weights were noted, and no significant hematologic abnormalities were found (Table 1). Although neutrophil and monocyte counts were slightly elevated in the treatment groups relative to the control group at 48 hours, the increased values remained within the normal reference range, and these values had recovered to control levels by 28 days and 63 days after injection (Table 1). This transient elevation most likely reflected local injection-related trauma resulting in local inflammation and necrosis of skeletal myofibers; thus we did not consider this an adverse effect but rather a normal physiologic response. The slight decrease in lymphocytes counts in the high-dose group versus control group at 48 hours after injection also remained within the normal reference range and likely reflected a stress response rather than a treatment-related adverse effect. In terms of statistically significant differences, the mean corpuscular volume of red blood cells had dropped at 48 hours in the high-dose group, and mean corpuscular hemoglobin concentrations had risen in the low-dose and reference-dose groups at 63 days relative to the control-group values (Table 1). None of these findings indicate a clinically meaningful adverse effect, because all differences remained within the normal reference range and the mean corpuscular volume had returned to control levels at 28 days after dosing.

Similarly, no significant abnormalities of serum chemistry tests were noted (Table 2). Mild elevations in aspartate transaminase (AST) and alkaline aminotransferase (ALT) were observed at all 3 time points in some groups, but they were not dose-related and the liver, heart and kidney were normal histologically. Rather, these slight variations in values reflected the combination of minimal injury to skeletal muscle and false increases from slight hemolysis of blood samples. Other statistically significant differences were noted in the increase of globulin level (*P*=0.014), and decrease of AST (*P*=0.05) and ALT (*P*=0.017) levels at 28 days in the reference group relative to the control group (Table 2), but these findings did not confirm a dose-related trend change and therefore were not considered clinically adverse effects.

### 3.2. Pathological Findings

No gross pathologic lesions were found during necropsy, and no histopathologic evidence of acute or chronic systemic toxicity or inflammation was observed in any of the tissues examined (including brain, spinal cord, lung, heart, liver, kidney, and spleen) from the control and high-dose groups at 48 hours or 28 days after i.m. injection of CoCl_2_:NAC.

**Figure 1:**
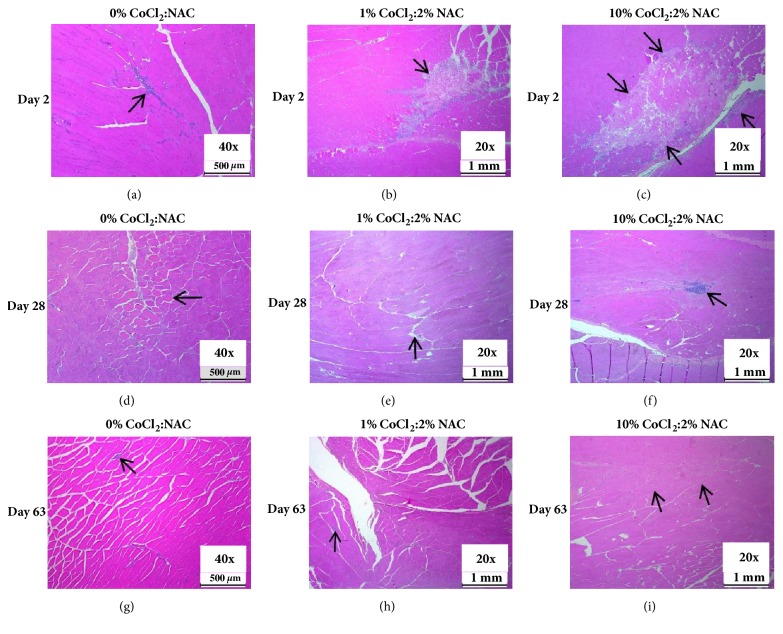
Histopathological changes of skeletal muscle at the site of C4 (CoCl_2_:NAC) injection. A volume of nine microliters of CoCl_2_:NAC at various concentrations (0% CoCl2:NAC [control], 1% CoCl2:2% NAC [mid- or reference dose], and 10% CoCl2:2% NAC [high dose]) was injected intramuscular into the middle region of the left hind limb quadriceps femoris muscle of male Sprague-Dawley rats. Images of H&E stained tissue sections at 20x and 40x magnification. (a)–(c) Images show mild inflammation (arrow) in control animals (a) and acute degeneration and necrosis of myofibers with granulomatous inflammation at the site of intramuscular injection (arrows) in the mid- and high-dose groups ((b) and (c), respectively), at 48 hours after treatment. (d)–(f) Images show minimal edema with occasional degenerated myofibers (arrow) in control (d) and middose group (e) animals, which is associated with focal mild lymphohistiocytic inflammation (arrow) and myofiber regeneration in the high-dose group (f), at 28 days after treatment. (g)–(i) Images reveal presence of rare small foci of histiocytic inflammation (arrow) similar in control and treated groups of animals ((g), (h), and (i)), and areas of normally regenerated myofibers surrounded by minimal interstitial fibrosis in the mid- and high-dose groups ((h) and (i), respectively), at 63 days after treatment.

Acute dose-related injury of transient focal myotoxicity at the injection site was noted in all three treatment groups at 48 hours after injection (Figures 1(a)–1(c)). Areas of affected muscle tissue were typically about 1 mm × 4 mm and showed acute degeneration and necrosis of myofibers of skeletal muscle. Four of the five mice in the reference-dose group and all five of the mice in the high-dose group were affected with this lesion. Moreover, the extent of the degeneration and necrosis of myofibers (measured with an Aperio whole-slide digital image scanner) was considerably greater in the high-dose group (average of the affected muscle area: 5,080,354 *μ*m^2^) than in the reference-dose group (average of the affected muscle area: 1,873,015 *μ*m^2^). Acute granulomatous inflammation with infiltration of neutrophils and macrophages at the injection site was observed in control and treated animals at 48 hours, and this was consistent with normal tissue reaction to injected material and early healing response. The severity of inflammation at the site of injection was associated with the dose level, indicating an acute local toxic effect of C4 to skeletal muscle (Figures 1(a)–1(c)).

In the chronic stages of treatment, at 28 and 63 days after injection, no degenerative or necrotic lesions were observed in the left quadriceps femoris in any animals from the control or treated groups (Figures 1(d)–1(f)). Indeed, skeletal muscle myofibers at the injection site had largely returned to normal by Day 28, albeit with scattered very small foci of macrophages (histiocytosis) and areas of regenerated myofibers, indicating resolution of the muscle lesions and associated minimal inflammation at the site of injection. No evidence of ongoing treatment-related tissue injury was noted at 63 days after treatment, when no microscopic or macroscopic lesions were observed in any of the control or treated groups (Figures 1(g)–1(i)). Further progression of tissue healing was reflected in the presence of smaller size regenerated myofibers surrounded by minimal interstitial fibrous tissue and occasional small foci of infiltrated histiocytes at the injection site.

## 4. Discussion

The results of this study of Sprague-Dawley rats indicated that a single intramuscular injection, in the middle of the quadriceps femoris muscle, of up to 10% CoCl_2_:2% NAC produced no significant treatment-related adverse clinical signs, no changes in body or organ weights, and no significant changes in hematologic or serum chemistry values. Similarly, postmortem pathological investigations revealed no gross or histopathologic evidence of acute or chronic systemic toxicity or inflammation. At 48 hours after i.m. injection, minor acute hematologic changes were noted in neutrophil, monocyte, and lymphocyte numbers, and mild focal myotoxicity manifested with acute degeneration and necrosis of myofibers associated with acute granulomatous inflammation at the injection site. Since these lesions healed and returned to normal skeletal muscle at 28 and 63 days after i.m. injection, these changes are considered to be a normal physiologic healing response to transient local acute muscle injury.

MRI provides superb anatomic resolution [8]; moreover, it can also visualize physiological changes in the tumor microenvironment or organ perfusion via dynamic contrast-enhanced MRI (DCE-MRI) [9, 10], which requires intravenous administration of gadolinium-based MR contrast agents [11]. Such agents are routinely used in clinical practice and were thought to be relatively safe [12]. However, gadolinium-enhanced MRI was recently linked with nephrogenic systemic fibrosis, a severe, potentially fatal adverse reaction, in some patients with renal impairment. This syndrome is a rare idiopathic skin condition, accompanied by pain and loss of mobility with involvement of the kidney and other organs such as lung, heart, liver, and diaphragm [12–16]. An alternative to gadolinium-based agents, C4, includes ionized cobalt [Co^2+^, or Co(II)], which can mimic hypoxia and subsequently generate reactive oxygen species (ROS) in cultured cells and induce direct cellular injury [17, 18]. Also, excessive generation of ROS may stimulate inflammatory processes [18]. Previous work has shown that complexing NAC with Co(II) seems to prevent cobalt-related toxicity in the prostate and periprostatic tissues after injection [7] and that C4 does not prompt excess ROS generation in human cancer cells or normal tongue cells [19]; nevertheless, little was known of the potential for acute or chronic systemic toxicity from C4* in vivo*. Here, we showed that a single intramuscular injection of C4 at a concentration 10 times than intended for clinical use, or 100 times more than what would be released from the spontaneous rupture of 120 MRI markers in a human prostate, led to no acute or chronic systemic toxicity or inflammation, no microscopic damage to brain, spinal cord, lung, heart, liver, kidney, or spleen, and no changes in the body or organ weights or in the clinical blood analyses. These findings suggest that C4 is not likely to cause acute or chronic systemic toxicity when in clinical use for prostate cancer brachytherapy.

With regard to acute local response, we did find evidence of focal acute degeneration and necrosis of myofibers associated with acute granulomatous inflammation at the injection site 48 hours after the i.m. injection. One review [20] showed similar histologic findings at 48 hours after an intramuscular injection of saline to rodents and rabbits, with evidence of myofiber regeneration and mild fibrosis at 10 days and 42 days after the injection. These findings are similar to observations in our study at 28 and 63 days after the injection, and they are also similar to those of another study involving intramuscular injections of antibiotics in sheep [21]. We consider these focal acute changes to be a normal, transient response to muscle trauma at 48 hours, the extent of which was similar to that from intramuscular injection of saline or antibiotics. Moreover, because the encapsulated C4 will be implanted in prostate tumors, we anticipate that this local injection response does not have the potential to harm patients.

Our study did have limitations. Not all of the biochemical markers that have been suggested as possible toxic indicators were included in this study, as sometimes it is impossible to plan for all measurements of toxicity when using small species.

## 5. Conclusions

In conclusion, we showed that a single intramuscular dose of the novel MRI-marker C4, in concentrations ranging from 0 to 10 times the dose intended for clinical use, produced no acute or chronic clinical systemic toxicity or inflammation in Sprague-Dawley rats. These results suggest that C4 may not cause acute or chronic clinical systemic toxicity or inflammation for clinical use in brachytherapy.

## Figures and Tables

**Table 1:** Hematologic findings.

**Time after Injection and Treatment Group**	**MCV, fL**	**MCHC, g/dL**	**Neutrophils**		**Monocytes**		**Lymphocytes**	
			**×10** ^**3**^/µ**L**	%	**×10** ^**3**^/µ**L**	%	**×10** ^**3**^/µ**L**	%
**Day 2**								
**Control**	61.7 (57.8-64.0)	30.8 (29.1-32.6)	2.3 (1.04-2.99)	18.4 (10.4-24.9)	0.27 (0.10-0.43)	2.1 (1.0-2.7)	9.63 (6.76-11.84)	76.6 (68.7-87.2)
**Low dose**	61.1 (57.1-63.3)	30.9 (29.5-32.1)	3.1 (1.67-4.63)	21.6 (15.3-27.9)	0.33 (0.25-0.56)	2.3 (1.8-2.8)	10.5 (8.43-15.38)	72.5 (66.7-80.9)
**Ref dose**	61.3 (57.8-62.9)	30.1 (29.4-30.6)	3.1 (0.9-5.27)	20.1 (11.2-26.7)	0.33 (0.09-0.54)	2.1 (1.2-2.8)	10.1 (6.84-15.13)	74.7 (67.0-85.4)
**High dose**	58.5*∗* (56.8-60.0)	30.9 (30.2-31.7)	3.5 (2.15-4.64)	26.6 (19.0-34.4)	0.4 (0.27-0.87)	2.9 (2.0-5.4)	8.7 (6.75-10.15)	66.7 (58-75.3)
**Day 28**								
**Control**	58.7 (58-59.6)	30.5 (30.3-30.7)	2.21 (1.03-2.87)	22.1 (11.6-28.4)	0.22 (0.16-0.3)	2.2 (1.5-2.9)	6.99 (6.31-7.39)	71.7 (62.4-82.9)
**Low dose**	59.3 (56.5-62.0)	30.5 (30.1-30.9)	2.5 (1.31-3.95)	21.6 (15-34.4)	0.27 (0.18-0.35)	2.5 (1.5-4.0)	8.3 (6.23-13.28)	71.6 (55.6-80)
**Ref dose**	58.8 (57.3-59.8)	31.1 (29.8-31.7)	2.3 (1.08-3.57)	24.2 (13.1-37.1)	0.31 (0.16-0.56)	31. (1.9-5.9)	6.8 (5.26-9.78)	69.4 (54.8-81.5)
**High dose**	58.8 (56.5-60.1)	30.6 (29.8-31.4)	2.7 (2.07-3.20)	23.1 (20.1-26.8)	0.31 (0.20-0.53)	2.7 (1.9-4.8)	8.2 (7.05-10.87)	70.4 (63.5-73.8)
**Day 63**								
**Control**	58.8 (57.7-61.2)	30.9 (30.6-31.4)	1.65 (0.95-2.29)	15 (8.9-20.3)	0.22 (0.16-0.37)	2 (1.3-3.3)	8.83 (7.43-9.96)	80.5 (73.2-87.0)
**Low dose**	58.9 (57.0-60.5)	31.9*∗* (31.0-32.5)	2.0 (1.16-2.52)	16.2 (12.0-19.4)	0.26 (0.19-0.35)	2.2 (1.5-3.1)	9.3 (8.03-10.75)	78.5 (74.7-83.6
**Ref dose**	58.7 (57.2-60.7)	32.2*∗* (32-32.5)	1.8 (1.06-2.68)	15.7 (10.1-24.2)	0.23 (0.11-0.34)	2.0 (1.3-3.2)	8.8 (7.01-11.99)	78.6 (69.6-85.3)
**High dose**	57.8 (55.8-61.1)	31.4 (30.1-32.3)	1.6 (1.0-2.38)	12.6 (6.9-16.8)	0.25 (0.09-0.38)	1.9 (0.9-2.7)	10.4 (9.13-12.91)	82.8 (78.0-89.9)

Data are shown as mean (range).*∗*Significantly different from control at *P*< 0.05.

Control, 0% CoCl_2_:NAC; Low dose, 0.1% CoCl_2_:2% NAC; Ref[erence] dose 1% CoCl_2_:2% NAC; High dose, 10% CoCl_2_:2% NAC.

MCV, mean corpuscular volume (in femtoliters); MCHC, mean corpuscular hemoglobin concentration.

**Table 2:** Clinical chemistry findings.

**Time after Injection and Treatment Group**	**Total Bilirubin,**	**Creatinine,**	**Blood Urea Nitrogen,**	**Aspartate Transaminase,**	**Alkaline Aminotransferase,**	**Alkaline Phosphatase,**	**Total Protein,**	**Albumin,**	**Globulin,**
	**mg/dL**	**mg/dL**	**mg/dL**	**mg/dL**	**IU/L**	**IU/L**	**g/dL**	**g/dL**	**g/dL**
**Day 2**									
**Control**	0.1 (0.1)	0.3 (0.28-0.32)	15.80 (14.2-16.7)	219 (122-445)	175 (54-441)	168 (98-228)	6.18 (5.58-6.59)	4.21 (4.01-4.60)	1.97 (1.57-2.30)
**Low dose**	0.1 (0.1)	0.3 (0.28-0.35)	16.40 (14.8-18.8)	283 (121-869)	266 (78-921)	208 (132-306)	6.23 (5.83-6.74)	4.16 (4.11-4.22)	2.07 (1.72-2.61)
**Ref dose**	0.1 (0.1)	0.27 (0.24-0.30)	16.9 (15.9-19.6)	199 (68-491)	173 (44-428)	201 (175-239)	6.1 (5.88-6.28)	4.2 (4.09-4.35)	1.9 (1.53-2.18)
**High dose**	0.1 (0.1)	0.29 (0.25-0.34)	16.1 (14.0-18.8)	132 (70-318)	99 (44-244)	167 (144-230)	6.21 (5.82-6.44)	4.04 (3.87-4.16)	2.17 (1.95-2.45)
**Day 28**									
**Control**	<0.1 (<0.01)	0.32 (0.28-0.35)	17.9 (16.2-19.5)	– (154->3836)†	– (48->4070)†	186 (141-252)	6.3 (5.98-6.95)	4.26 (4.17-4.47)	2.04 (1.78-2.48)
**Low dose**	<0.01 (<0.01)	0.31 (0.27-0.34)	20.1 (17.3-24.6)	– (123->964)†	– (65->921)†	202 (173-258)	6.36 (5.91-6.74)	4.18 (3.95-4.36)	2.17 (1.76-2.66)
**Ref dose**	<0.01 (<0.01)	0.32 (0.29-0.37)	12.2 (15.8-20.2)	– (56->2530)†	– (45->3030)†	140*∗* (128-163)	6.68 (6.34-6.83	4.19 (4.04-4.31)	2.49*∗* (2.30-2.65)
**High dose**	<0.01 (<0.01)	0.31 (0.31->0.69)†	19.3 (17.4-21.4)	– (59->7000)†	– (45->7000)†	210 (131-287)	6.87 (6.39-7.87)	4.32 (4.11-4.60)	2.51 (2.13-3.27)
**Day 63**									
**Control**	0.1 (0.1)	0.34 (0.32-0.36)	19.9 (18.8-21.4)	135 (68-325)	141 (41-473)	141 (111-195)	6.89 (6.56-7.21)	4.33 (4.21-4.44)	2.56 (2.21-2.88)
**Low dose**	0.1 (0.1)	0.33 (0.31-0.35)	20.7 (17.6-23.6)	142 (84-266)	113 (52-239)	165 (127-206)	7.01 (6.69-7.70)	4.40 (4.14-4.90)	2.61 (2.48-2.80)
**Ref dose**	0.1 (0.1)	0.32 (0.30-0.35)	19.6 (17.9-21.7)	247 (180-336)	193 (153-230)	149 (89-235)	6.57 (6.26-7.10)	4.22 (3.98-4.58)	2.36 (2.00-2.52)
**High dose**	0.1 (0.1)	0.31 (0.26-0.33)	18.4 (16.9-19.5)	136 (67-232)	114 (39-233)	115 (100-135)	6.73 (6.27-7.33)	4.16 (3.89-4.57)	2.57 (2.38-3.07)

Data are shown as mean (range). *∗*Significantly different from control at *P*< 0.05. †Out of range because of hemolysis.

Control, 0% CoCl_2_:NAC; Low dose, 0.1% CoCl_2_:2% NAC; Ref[erence] dose, 1% CoCl_2_:2% NAC; High dose, 10% CoCL_2_: 2% NAC.

## Data Availability

The data used to support the findings of this study are available from the corresponding author upon request.

## Supplementary Materials

Supplementary Materials Supplemental Table 1: measurement of free cobalt in C4 solution incubated at 37°C. Supplemental Table 2: measurement of free cobalt from C4 solution in human salvage plasma. Supplemental Table 3: tissues collected from rats at necropsy at 48 hours, 28 days, or 63 days after a single intramuscular dose of C4.
