# Supplementary material for: Toxicity Evaluation of a Novel Magnetic Resonance Imaging Marker, CoCl2-N-Acetylcysteine, in Rats
Source: J Toxicol. 2018 Dec 2;2018:9173452. doi: 10.1155/2018/9173452 (PMC6304599; doi:10.1155/2018/9173452)
Supplement: Supplementary Materials — Supplemental Table 1: measurement of free cobalt in C4 solution incubated at 37°C. Supplemental Table 2: measurement of free cobalt from C4 solution in human salvage plasma. Supplemental Table 3: tissues collected from rats at necropsy at 48 hours, 28 days, or 63 days after a single intramuscular dose of C4. [file 9173452.f1.docx]

**Supplemental Table 1**. **Measurement of free cobalt in C4 solution incubated at 37 ^o^C**

Duration of incubation (Hours) Percentage of free cobalt (%)

0 5.5 ± 0.2

48 6.5 ± 0.5

96 8.8 ± 0.4

120 7.0 ± 0.0

**Supplemental Table 2**. **Measurement of free cobalt from C4 solution in human salvage plasma**

Duration of incubation (Hours) Percentage of free cobalt (%)

0 0.8 ± 0.8

48 5.3 ± 0.8

**Supplemental Table 3**. **Tissues collected from rats at necropsy at 48 hours, 28 days, or 63 days after a single intramuscular dose of C4**

Digestive System Endocrine System

Liver* Pituitary gland

Esophagus Thyroid gland

Stomach Parathyroid glands

Duodenum Adrenal glands

Jejunum

Ileum Nervous System

Cecum Brain (including cerebrum, cerebellum, medulla/pons)

Colon Spinal cord (cervical, thoracic, lumbar)

Salivary glands

Pancreas Musculoskeletal System

  Skeletal muscle

Urinary System Femur/knee joint

Kidneys* Sternum

Urinary Bladder

  Miscellaneous

Respiratory System Skin

Lungs Eyes

Trachea Gross observations^‡^

Larynx Injection site

Pharynx

Nasal Cavity Reproductive System

  Testes

Cardiovascular System Epididymides

Heart Prostate

Aorta Seminal Vesicles

Hematopoietic System

Spleen*

Thymus

Mandibular lymph node

Mesenteric lymph node

Bone marrow^†^

*These organs were weighed at necropsy

^†^Bone marrow assessed with the sternum.

^‡^This describes observations for which histopathologic analysis was not appropriate (e.g., fluid, ruffled fur, and missing anatomic parts)
